# Flexible Electrode Based on MWCNT Embedded in a Cross-Linked Acrylamide/Alginate Blend: Conductivity vs. Stretching

**DOI:** 10.3390/polym12010181

**Published:** 2020-01-09

**Authors:** Jake Thibodeau, Anna Ignaszak

**Affiliations:** Department of Chemistry, University of New Brunswick, Fredericton, NB E3B 5A3, Canada; n9tqn@unb.ca

**Keywords:** flexible electrode, cross-linked acrylamide/alginate, carbon nanotubes, tensile strength, impedance spectroscopy

## Abstract

A polyacrylamide-alginate hydrogel electrolyte, blended with Multi-Walled Carbon Nanotubes (MWCNT) as an electronically conductive fraction, allows for the creation of a flexible, durable, and resilient electrode. The MWCNT content is correlated with mechanical characteristics such as stretch modulus, tensile resistance, and electrical conductivity. The mechanical analysis demonstrates tensile strength that is comparable to similar hydrogels reported in the literature, with increasing strength for MWCNT-embedded hydrogels. The impedance spectroscopy reveals that the total resistance of electrodes decreases with increasing MWCNT content upon elongation and that bending and twisting do not obstruct their conductivity. The MWCNT-inserted hydrogels show mixed ionic and electronic conductivities, both within a range of 1–4 × 10^−2^ S cm^−1^ in a steady state. In addition, the thermal stability of these materials increases with incrementing MWCNT content. This observation agrees with long-term charge-discharge cycling that shows enhanced electrochemical durability of the MWCNT-hydrogel hybrid when compared to pure hydrogel electrolyte. The hydrogel-carbon films demonstrate an increased interfacial double-layer current at a high MWCNT content (giving an area-specific capacitance of ~30 mF cm^−2^ at 2.79 wt.% of MWCNT), which makes them promising candidates as printable and flexible electrodes for lightweight energy storage applications. The maximum content of MWCNT within the polymer electrolyte was estimated at 2.79 wt.%, giving a very elastic polymer electrode with good electrical characteristics.

## 1. Introduction

Advances in hydrogel performance, when combined with interstitial conductive materials, have shown potential for incorporation with modern electronic applications [1,2]. Technological products, such as wearable electronics [3,4], personal sensors [5,6,7], and smart textiles [8,9], are examples where these novel materials can enhance their functionality. Successful implementation requires materials that are durable, flexible, and resilient, capable of meeting the conductivity and physical requirements. Modularity is a central asset, as many modern devices currently are rigid and bulky. Innovations may allow for new materials and devices that are able to conform to human mobility demands.

These electronics can be utilized either in an active or passive way. An active device responds to user input; for example, as two-way radio communications or live GPS data transmissions to emergency responders [10,11]. Passive devices do not require user input, but rather provide sensory data or function to the user, or to an external support point [12]. Examples include providing vital signs to medical staff (such as the Georgia Tech Wearable Motherboard, or Smart Shirt) [13,14], body armor cooling devices by police and military [15], or smart GPS tracking for the elderly suffering from dementia [16]. The applications can also be implemented into personal electronic devices—smartwatches and fitness trackers are increasingly common in everyday usage [17]. Additionally, new lightweight energy materials are highly sought after in applications such as exoskeletons for physical rehabilitation [18], biomimetic actuators [19], field-effect transistors [20], and chemical sensing for air pollution and other biohazards [21].

For this research, in order to determine a suitable basis for composition, we examined an acrylamide-alginate flexible material pioneered by Sun et al. [22]. Polymers, with the applications in mind, must endure various physical stresses, such as stretching, twisting, and bending. The double-network structure made of cross-linked polymers results in a greater fracture strength compared to single networks, with high retention of shape against temporary mechanical deformations [23]. This is attributed to the combined interactions of short-chain polymers providing mechanical strength, and long-chain polymers providing elasticity. Previous research in this area shows that short-chain polymers cross-linked covalently suffer permanent damage upon deformation of the material [22]. However, a known method of minimizing this unwanted effect is the replacement of the covalent cross-linking bonds with non-covalent (i.e., ionic) using multivalent ion coordinators [24]. This allows a formation of much weaker ionic linkage between the rigid polymer blocks, observed as reduced stiffness of the gel, and thus resulting in more stretchable material. Among multivalent cations that generated suitable ionic cross-linking were Ca^2+^, Sr^2+^, Ba^2+^, Al^3+^, and Fe^3+^ [25]. Calcium was chosen due to the relative minor environmental impact that it has upon degradation, along with a relatively strong ionic coordination strength. Initial formulations were based on similar work by Demianenko et al. [26], in part due to the utilization of UV irradiation in the curing process that shortens the synthesis time.

Multi-Walled Carbon Nanotubes (MWCNTs) were chosen as the electron-conducting component of the flexible electrode proposed in this work. MWCNT-alginate hydrogels have been previously explored by Joddar et al. [27], with a focus on mechanical properties—notably with overall declining performance above 1 mg/mL of MWCNT (to a tested max of 5 mg/mL). Hong et al. [28] integrated single-walled CNTs in poly (dimethyl siloxane) and analyzed their electrical characteristics in various circuits, as well as under differing mechanical strain. Sudha et al. [29] created an MWCNT-polyacrylamide hydrogel, exploring dehydration and rehydration properties, with a brief examination of their conductivities. Among other carbon allotropes, carbon nanotubes are particularly researched as they show several benefits when combined with various polymer electrolytes. CNTs not only facilitate good electronic conductivity, but also improve the overall mechanical strength and thermal stability of these hybrid materials [30]. In addition, they are excellent double-layer capacitors that cannot be outperformed by more affordable carbons. Thus, CNTs are of interest in energy harvesting and reposition applications [28,31].

In this study, we have investigated the conductivity and mechanical features of a polyacrylamide-alginate hydrogel electrolyte, combined with MWCNTs as the electronically conductive component. The effect of MWCNT content in the flexible electrode is verified by mechanical characteristics such as stretch modulus and tensile resistance. In addition, electrochemical characteristics such as conductivity, long-term electrochemical stability upon charging, potential stability, and thermal stability were examined. Mechanical impact and deformation on conductivity were analyzed by impedance spectroscopy upon elongation, bending, and twisting of the hydrogel electrolyte and MWCNT-embedded electrodes. On the macroscopic level, we determined the maximum loading of MWCNT within the hydrogel with an overall aim to create an electrode that has the flexibility to endure moderate strains and stress. Ultimately, this material can be used as a conducting platform in applications such as smart textiles, personal sensors, wearable electronics, and lightweight energy storage and conversion.

## 2. Materials and Methods

Materials: Calcium chloride dihydrate (99.0–105.0%) was purchased from Fisher Scientific (Ottawa, ON, Canada). Multi-walled carbon nanotube (thin and short, <5% metal oxide), potassium chloride (99.6%), *N*,*N*′-methylenebisacrylamide powder (≥99.5%), acrylamide powder (≥99%, HPLC grade), alginic acid sodium salt powder, *N*,*N*,*N*′,*N*′-tetramethylethylenediamine (≥99%, GC grade), and ammonium persulfate (≥98%) were obtained from Sigma Aldrich (Oakville, ON, Canada). Ultra-pure distilled water (18 MΩ) was used as the reaction solvent. Fluorine-doped tin oxide (FTO) glass with a surface resistivity of 7 Ω (Sigma Aldrich, Oakville, ON, Canada) was cut into 2.5 cm × 5.5 cm × 0.2 cm slides, acting as the current collector in electrochemical tests, and layered with copper foil at the electrode clip contact area.

Methods: Tensile tests were performed using an Instron 4465 Tensile test workstation equipped with 1 kN of load cell under air. The elongation rate was kept as 40 mm min^−1^. Thermogravimetry analysis (TGA) was carried out on the TA Instruments analyzer model Q50 (TA Instruments, Mississauga, ON, Canada). The mass of samples ranged from 9.0 to 10.3 mg. N_2_ gas was kept at the flow rate of 90 mL min^−1^. A ramp temperature was 10 °C min^−1^ over the range from 25 to 600 °C. Fourier-transform infrared (FTIR) analysis was carried out using a Bruker spectrometer model ALPHAII with a single reflection ART diamond accessory (Bruker, Billerica, MA, USA). All hydrogels were dehydrated prior to TGA and FTIR tests by drying at 70 °C for 24 h in a laboratory oven.

Electrochemical tests were carried out using a CH Instruments electrochemical workstation (model CHI660E, CH Instruments Inc., Austin, TX, USA). All electrochemical characteristics were done using a two-electrode cell consisting of two FTO plates used as a current collector. The samples were square shape with a 12 mm edge, with a typical thickness of 4.5 mm (varying slightly based on the addition of MWCNT), and mounted between FTO plates. For the conductivity test upon elongation, one of the FTO plates was moved with a distance increment of 1 cm and the length and the thickness of the sample were measured using a caliper. The reported conductivity of materials is as-obtained (in siemens, S) and with the correction to the size (thickness, length, and width) of the stretched sample (in S cm^−1^).

Cyclic voltammetry was acquired at a scan rate varying from 0.05 to 0.2 V s^−1^ and from −0.8 V to 0.8 V in the two-electrode cell (without a reference). Impedance spectroscopy was carried out in the frequency range of 1 × 10^5^–1 Hz, with an amplitude of 0.005 V, and at the constant polarization of 0 V. ZView software (Scribner Associates Inc., Southern Pines, NC, USA) was used for the modeling of impedance spectra. The electrochemical stability was carried out by applying a 2500 scan of the charge-discharge at 0.5 mA current load, with a cathodic and anodic time of 30 s and (zero hold time giving a total time of the degradation test at 21 h). The charge-discharge was also carried out at 0.005, 0.01, 0.05, 0.1, 0.25, 0.5, 0.75, and 1.0 mA for all samples, with preliminary extended testing for pure/360 mg from 0.005 mA to 2.5 mA. However, changes at the interface material-current collector (assumed as beginning of degradation) were observed at 0.5 mA, which was chosen for the long-term durability test.

Synthesis of Hydrogel Electrolyte and Electrode: Ten milliliters of deionized water (that is a constant volume of water used for each hydrogel formulation) was purged with dry nitrogen for 30 min prior to use. Then, 1.4470 g of acrylamide, 0.7455 g of potassium chloride, and 0.0016 g of *N*,*N*′-methylenebisacrylamide were dissolved in 4 mL of deionized water. Separately, 0.1809 g of sodium alginate was dissolved in 3 mL of deionized water and combined with the first mixture under vigorous stirring. Furthermore, for the MWCNT-embedded electrodes, the designated amount of MWCNTs, was added (0.010–0.360 g) upon stirring. Afterward, 4.6 µL of *N*,*N*,*N*′,*N*′-tetramethylethylenediamine was injected into this mixture. In a separate flask, 0.0435 g of ammonium persulfate and 0.0927 g of calcium chloride dihydrate were dissolved in 3 mL of water, combined with the remaining reactants upon stirring, poured onto a petri dish (60 mm diameter), and placed in a UV cross-linker for 60 min of irradiation at a power of 300.000 μJ cm^−2^ (254 nm, model VWR^®^ UV Crosslinker, VWR, Ville Mont-Royal, QC, Canada). All procedures were carried out under ambient conditions. The MWCNT content was varied resulting in the following formulations (names of hydrogels correspond to the mg of MWCNT): 40 mg refers to MWCNT concentration of 4 mg mL^−1^ (mL of water), that is 0.32 wt.% of MWCNT; 80 mg contains 8 mg mL^−1^ giving 0.63 wt.% of MWCNT; 120 mg has 12 mg mL^−1^ corresponding to 0.95 wt.% MWCNT; 240 mg has 24 mg mL^−1^, that is 1.88 wt.% MWCNT; 360 mg has 36 mg mL^−1^, that is 2.79 wt.% MWCNT; and 480 mg has 48 mg mL^−1^, giving 3.69 wt.% MWCNT.

## 3. Results and Discussion

### 3.1. Structure and Thermal Stability: Effect of MWCNT Content on Interactions between Molecular Components and Morphology

**Figure 1:**
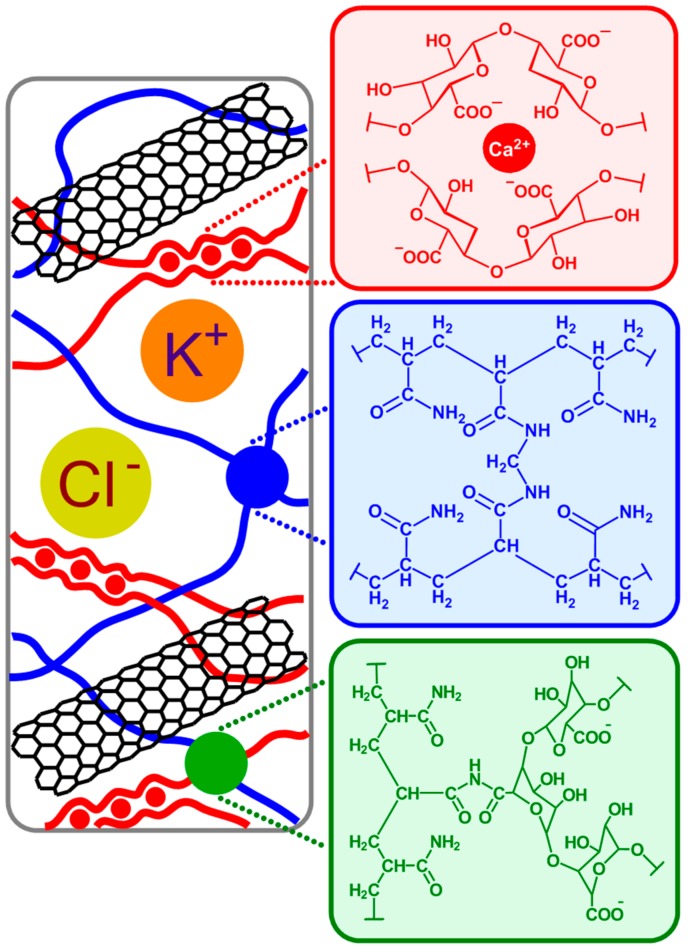
Structure of a Multi-Walled Carbon Nanotubes (MWCNT)-embedded hydrogel consisting of an alginate blocks formed via ionic cross-linking through Ca^2+^ (red); polyacrylamide blocks derived from covalent crosslinks through *N*,*N*′-methylenebisacrylamide (blue); alginate-polyacrylamide hybrid gel fraction: the two types of polymer network are joined by covalent crosslinks (green).

The hydrogel matrix proposed in this work is formed via copolymerization of acrylamide and bis-acrylamide, with the optimal composition decided based on work reported by Sun et al. [22]*,* and by a previous study carried out in our group [31]. Briefly, the reaction is vinyl addition polymerization via a free radical-generating system initiated by ammonium persulfate and TEMED. TEMED accelerates the rate of formation of radicals from persulfate that reacts with acrylamide monomers (resulting in radicals that react with neutral monomer and form a polymer). The extending polymer chains are randomly cross-linked by *N*,*N*′-methylenebisacrylamide (Figure 1; blue), resulting in stiff gels. In addition, in the presence of alginate and calcium chloride, two types of cross-linked polymer blocks are formed: ionically cross-linked alginate (Figure 1; red), and covalently cross-linked polyacrylamide. In an aqueous solution, guluronic acid units in alginate chains form ionic crosslinks through Ca^2+^ coordination. By contrast, in a polyacrylamide hydrogel, the polyacrylamide chains form a network by covalent crosslinks. There also exist alginate-polyacrylamide hybrid fractions formed when the two types of polymer networks are joined by covalent crosslinks (Figure 1; green).

The most problematic aspect of these combined materials is the homogenous incorporation of MWCNT within the polymer. This is because of the tendency of MWCNT to agglomerate under electrostatic forces. Magnetic stirring, sonication, and other homogenization methods are not effective. Using surfactants for MWCNT dispersion in the solution of monomers is more challenging, affects the rate of polymerization, and ultimately the chemical composition of hydrogels, their mechanical strength, and conductivity. As compared to our previous study [31], Nafion, acting as a surfactant in an aqueous suspension of MWCNT, has been replaced with the viscous solution of sodium alginate. The alginate can both participate in the cross-linking and stabilize the MWCNT in water. Based on the chemical structure of alginate, we can assume that improvement of MWCNT dispersion is related to the modification of the electrical surface charge of MWCNT, defined as zeta-potential (unlike Nafion, which is composed of both hydrophobic and hydrophilic fractions that are typical for the surfactant structure) [30]. This potential is often related to an electric surface potential (*E_Elec_*) and contributes to the total surface energy, *E_tot_* = *E_VW_* + *E_Elec_*. *E_tot_* depends on the sum of attractive and repulsive forces between particles, which are both dependent on an inter-particle distance. The London-van der Waals contribution for two particles of the same material (*E_VW_*) is always attractive, thus promoting the aggregation of suspended particles. On the contrary, the repulsive component, *E_Elec_*, is related to the formation of an electric double-layer on the particle surface when immersed in a polar solvent. For example, in an aqueous suspension of functionalized MWCNT, negative charge is developed on the particle surface during ionization of the oxygen-rich functionalities (e.g., -COOH). Because of the surface charge, an electrostatic potential is created in the proximity of the nanoparticle, and a concentrated layer of counter ions, known as the Stern layer, is formed. As a result, the zeta potential at the MWCNT surface exceeds the total interaction potential barrier (*E_tot_*), and at a given distance, the particle repulsion is stronger and more attractive than the van der Waals forces, resulting in the formation of homogeneous suspensions [32,33]. With this, the carbon nanotubes could be well-dispersed even at very high content without the addition of Nafion.

**Figure 2:**
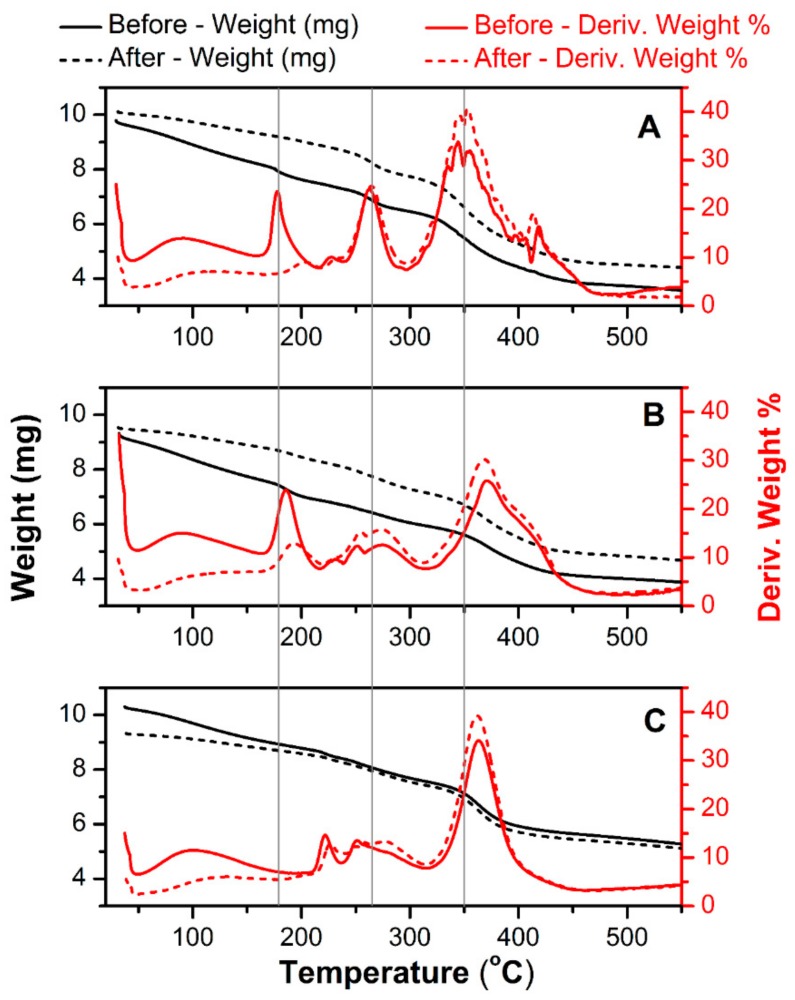
TGA (black) and first derivative of mass loss (red) of pure hydrogel electrolyte (**A**) and with 80 mg of MWCNT (**B**) and 360 mg (**C**). Solid lines refer to freshly prepared samples and dashed lines refer to samples after long-term electrochemical degradation using charge-discharge techniques.

Figure 2 shows a thermal gravimetric scan of a pure hydrogel (A) and an MWCNT-reinforced hydrogel at two differing MWCNT contents (8 mg mL^−1^ and 36 mg mL^−1^) plotted with the first derivative of weight loss (in %) as the function of applied temperature. The same tests were done for newly-prepared materials and the same films after a long-term electrochemical charge-discharge scanning (accelerated degradation). The latest was used to analyze possible chemical changes related to electrochemical degradation. All signals acquired below 200 °C are related to the removal of residual water. Regarding this, the hydrogel with the highest MWCNT content (Figure 2C) showed a negligible amount of water after drying. This is related to the fact that a significant weight fraction of this material is occupied by MWCNT, not by hydrophilic polymers. For the pure hydrogel (Figure 2A), an initial decomposition temperature (IDT) is detected at 221 °C and the final decomposition temperature (FDT) at 417 °C, resulting in 35% weight loss. The thermal degradation of polyacrylamide occurs in three pyrolysis phases. The first signal at IDT is attributed to the degradation of the pure and not cross-linked acrylamide monomer. Afterward, the decomposition of non-cross-linked polyacrylamide has a peak maximum at 264 °C. The main decomposition of the polyacrylamide cross-linked with *N*,*N*′-methylenebisacrylamide showed an onset temperature at 305 °C and endset at 440 °C. In the temperature range 221–310 °C, one ammonia molecule is liberated for every two amide groups, resulting in the formation of imide [22]. Subsequently, thermal degradation of imides and breaking of the polymer backbone occurs at higher temperatures. As discussed in previous work [22], pure alginate (not cross-linked with acrylamides) has two pyrolysis stages: the first thermal degradation process takes place in the temperature range 225–300 °C. The weight loss in the first stage is attributed to the degradation of the carboxyl groups (leading to CO_2_ release). The second stage occurred at much higher temperatures (650–740 °C) and corresponds to depolymerization resulting in a carbonaceous residue. Since the first step of decomposition of alginate overlap with signals related to acrylamide monomers, chemical analysis from the degradation of hybrid gels is difficult. One can observe that the pure alginate-polyacrylamide hybrid gel (Figure 2A) clearly shows pyrolysis stages shifted from locations of single networks [22]. This qualitatively demonstrates the formation of new covalent bonds between alginate and polyacrylamide.

MWCNT-embedded hydrogels (Figure 2B,C) exhibit improved thermal stability in comparison to pure hydrogels. This is manifested by the shift of decomposition temperature of the main polymer blend from 350 °C (Figure 2A—pure hydrogel) to 370 °C. The percent weight loss also decreases with increasing MWCNT content, especially for the highest carbon loading (Figure 2C). The higher resistance to heat in the presence of the MWCNT reinforced within the polyacrylamide/alginate network can be also related to weak chemical interactions between the MWCNT and the different reaction sites of both polymers [27,29]. For example, the latest study suggested that carboxyl groups present on the MWCNT surface can react with hydroxyl functionalities of alginate, resulting in hydrogen bridging [27]. These molecular interactions are believed to contribute to both better thermal stability of the MWCNT/polymer hybrid, and also to improved dispersion of the MWCNT in the solution of monomers and within the hydrogel itself. More probably, this chemical interaction is based on the esterification reaction and involves the mentioned carboxylic moieties from oxidized carbon and the hydroxyl groups of alginates. Nevertheless, this reactivity requires significant oxidization of the MWCNT surface (high content of -COOH), which was not found in the present study. The surface elemental analysis of MWCNT using an X-ray photoelectron did not show a significant difference in oxygen content (related to -COOH, -OH, or other oxygen-containing functional groups on the MWCNT surface; data not included) for as-obtained (unwashed) and the acid-washed MWCNT (3 M HNO_3_, reflux 1 h). Thus, improved dispersion of carbon within the polymer observed in this work can be assumed as the combined effects of electrostatic interactions (predominant) and a minor contribution from the chemical bond formation between alginate and carboxylic groups present on the MWCNT surface.

**Figure 3:**
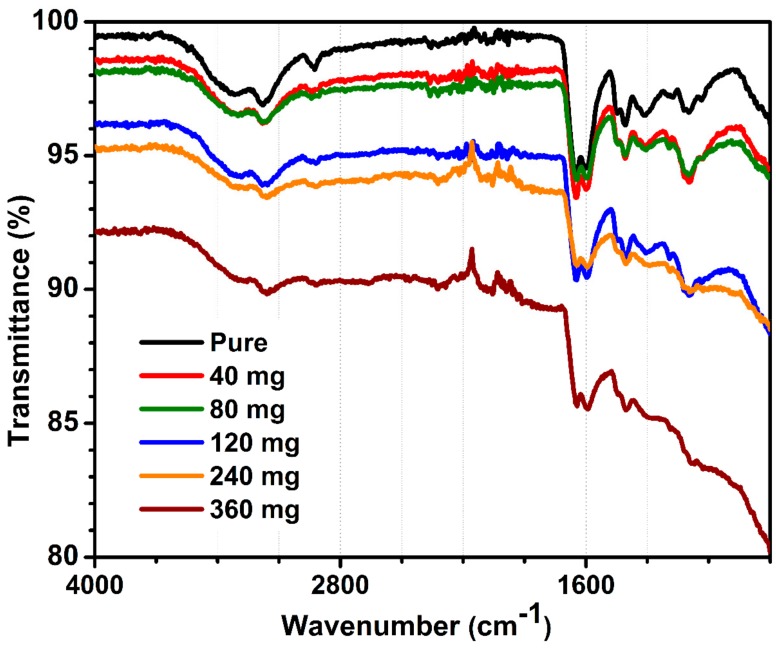
FTIR of pure and MWCNT-embedded hydrogels.

Figure 3 demonstrates the FTIR spectrum of pure and MWCNT-containing hydrogels with distinctive signals assigned to acrylamide and alginate polymers. The FTIR spectra for all MWCNT-embedded hydrogels were similar, and corresponded to hydrogel signals, except an intensity of peaks decreased with increasing MWCNT content. FTIR showed only signals related to polymers, which were not influenced by the presence of MWCNT at any loading. Briefly, peaks at 1113 and 1350 cm^−1^ corresponded to the C-O stretch and at 1181 cm^−1^ were assigned to the C-N stretch, together with the N-H vibration at 2940 cm^−1^. The vibrations at 1316, 1401, 1462, 2762, and 2855 cm^−1^ corresponded to the C-H bonding, arising from amines and amides present in the hydrogel structure. The band at 1606 cm^−1^ was related to the C=C vibration and the band at 1679 cm^−1^ was a signal of unreacted carbon double bonds from acrylamide and bis-acrylamide monomers. The amide stretch was also observed at 3442 cm^−1^ and the -OH stretch at 3178 cm^−1^. In summary, FTIR did not demonstrate chemical interactions between MWCNT and organic fraction. This is presumably due to a very low carbon content per total hydrogel mass, calc. 2.79 wt.% of MWCNT for hydrogels containing 360 mg of carbon.

### 3.2. Resilience—Mechanical Analysis

**Figure 4:**
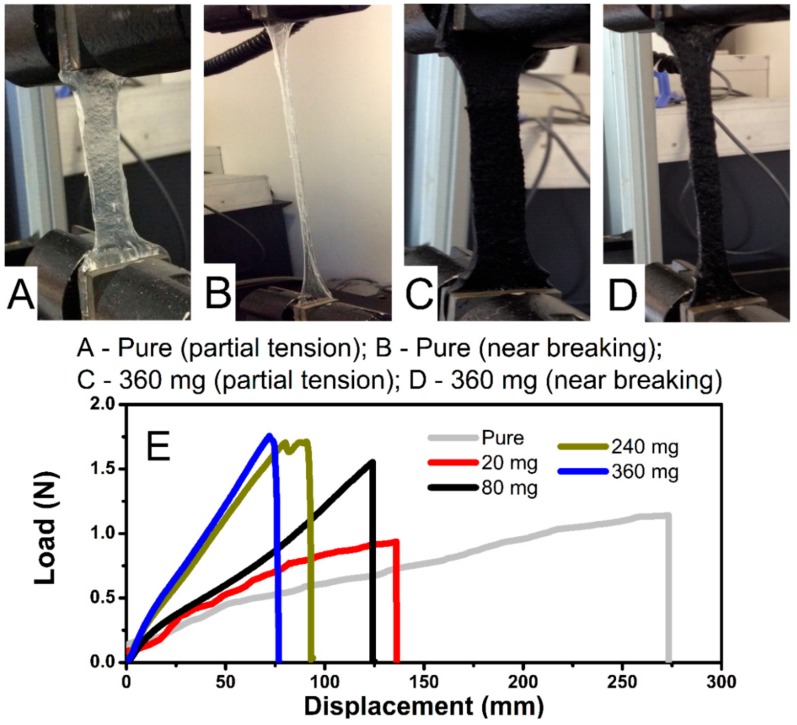
Mechanical tensile strength measurement set-up, showing pure sample under partial mechanical load (**A**) and near failure point (**B**). A 360 mg sample with partial mechanical load (**C**) and near failure point (**D**). Force loading and related displacement recorded during tensile measurements (**E**).

Tensile characteristics of pure hydrogel and its combinations with MWCNT are shown in Figure 4. They were used to explore a maximum elongation length before mechanical failure (Figure 4A–D) and tensile strength acquired from the load sensor attached to one end of the assembly (Figure 4, bottom). With an adjustment for area difference (thickness of films was relatively similar for all samples), pure hydrogel tested at a maximum of 22.3 kPa (1.34 N), at a displacement of 271 mm. For the hydrogel with the highest MWCNT content (36 mg mL^−1^), the applied sensor load was 28.8 kPa (1.76 N), at the displacement of 77 mm. Overall, the stretch modulus is sixteen times higher than that of the initial length for pure, and almost five times for MWCNT-embedded samples. In trend, an increase of MWCNT correlates to a general increase in tensile strength, with a more rapid decline in stretch modulus. A total force resistance of electrodes synthesized in this work was lower, yet resilience to deformation and stretch modulus held similar (17–21 factor) as compared to pure hydrogel with a similar composition as reported by others [22]. Although hydrogels fabricated in this work demonstrated lower force resistance to elongation, the comparison with the referred literature can be only qualitative since the results strongly depend on the thickness and geometry of the sample [22], the size of mounting clamps, as well as on the quality of the clamp (e.g., clamps with a sharp, uneven edge cause the material to be cut through faster). Joddar and colleagues reported the MWCNT embedded in alginate hydrogel (without acrylamide) at a very low load content [29]. The resilience ranges from 103.5 kPa for the pure hydrogel, 79.5 kPa (1 mg mL^−1^ MWCNT), 62 (3 mg mL^−1^ MWCNT), and 18 kPa (5 mg mL^−1^ MWCNT), with the decrease of total strength and stretch modulus with increasing MWCNT content observed. This conclusion contradicted their early central hypothesis that increasing the MWCNT content would increase the stiffness of the alginate gel [27]. This could be related to the weak van der Waals forces occurring between the MWCNT and alginate [27]. An opposite effect (increase in stiffness with increasing MWCNT content, Figure 4) is observed in this work. For hydrogels proposed in this work, the role of acrylamide blocks can improve resilience by additional alginate-acrylamide crosslinks (Figure 1), as well as the weak interactions (possibly hydrogen bringing) between alginate and acrylamide blocks with oxygen-containing functionalities present on the MWCNT surface. This suggests the participation of MWCNT strands within the polymer network.

As indicated in Figure 4E, there is a clear correlation between an increase in MWCNT content and an increase in tensile strength. Notably, the total MWCNT content of 36 mg mL^−1^ showed both the best mechanical and electrochemical characteristics. Further testing at 48 mg mL^−1^ indicated an oversaturation of the gel matrix, leading to unincorporated deposits of MWCNT on the surface after cross-linking was complete. The important consideration from this is the retention of physical properties of the hydrogel, with the addition of the MWCNT. Stretch was significantly reduced but retains enough capacity to serve practical applicability. An additional benefit is an increase in overall tensile force, indicating the contribution of MWCNT in the improvement of mechanical strength of hydrogels proposed in this work.

### 3.3. Impedance Analysis

**Figure 5:**
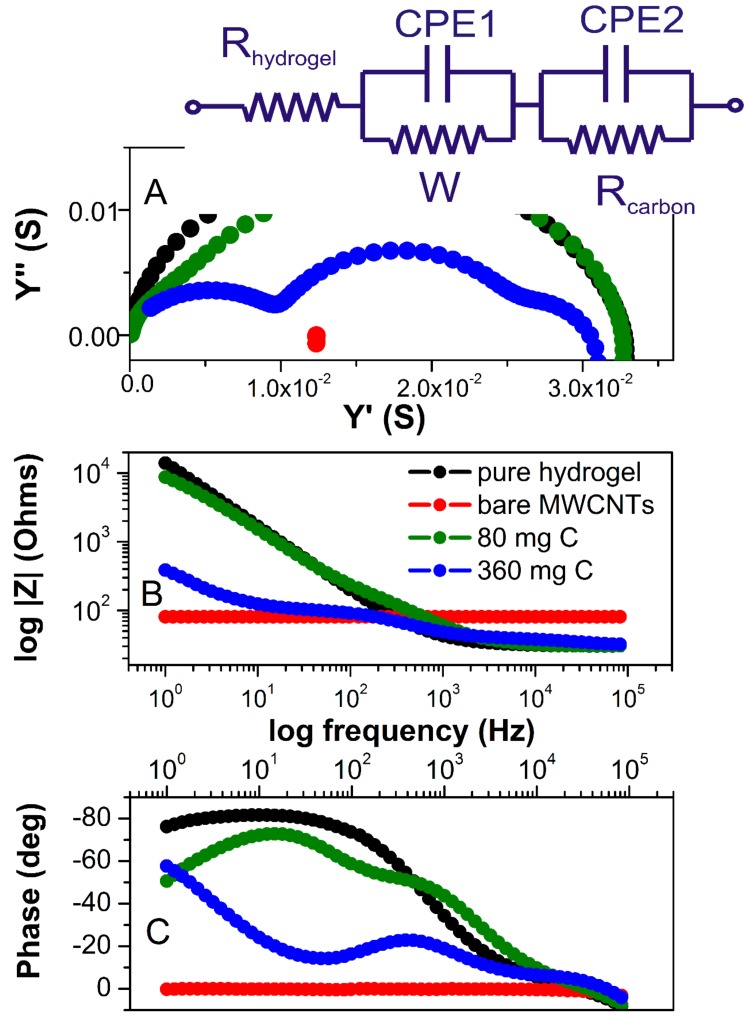
(**A**) Admittance spectra of MWCNT (red), pure hydrogel electrolyte (black), hydrogel with 80 mg MWCNT (green), and with 360 mg of MWCNT (blue). (**B**) Bode diagram of absolute impedance and (**C**) phase angle as the function of frequency. An electrical equivalent circuit representing the distribution of resistances and capacitances in the electrode was used for the fitting of the impedance spectra and is shown as an insert (R_hydrogel_ represents a resistivity of pure hydrogel; CPE1 is a pseudo-capacitance of the hydrogel; *W* is a Warburg resistor related to diffusion of ions within hydrogel electrolyte; R_carbon_ and CPE2 are resistance and capacitance of the carbon fraction, respectively).

For the polarizable electrode in the absence of redox reactions (non-faradaic measurements), the impedance signal will be dominated by the surface capacitance. This is true at least if the capacitance is low and the value of electrolyte solution is not too high. Given that electrodes in our study are symmetric, we can assume that the impedance between the electrodes can be modeled by electrolyte resistors and interfacial capacitance (Figure 5A). The impedance spectra of dry MWCNTs showed pure resistive behavior as represented by an absolute resistance that is independent of the frequency shown in the Bode diagram (red line in Figure 5B) and the phase shift remains at 0° in the whole frequency range (Figure 5C).

There are three distinct regions observed in the Bode plots for pure hydrogel (black spectrum). At the highest frequencies (between 10^5^ and 10^4^ Hz), resistive behavior is observed where the phase shift passes through 0° and the impedance magnitude is a horizontal line (because resistive impedance is not frequency-dependent). This represents the bulk resistance of the gel/electrolyte, which is used to calculate the hydrogel conductivities. In a broad range of frequency (800–1 Hz), capacitive behavior is observed as indicated by the negative |Z| slope (Figure 5B) and the phase angle approaching −90° (Figure 5C), which is due to double-layer capacitance formed at the interface of the electrolyte and FTO plates, and is typically observed in this frequency range. From the narrow range (400–800 Hz), diffusion behavior is observed as the phase angle approaches −45° (and the slope of impedance magnitude has changed). This represents the diffusion of ions through the finite thickness of the hydrogel. Considering all these components, the hydrogel EIS spectra can be fitted using an equivalent circuit shown in Figure 5A as an insert. Hydrogels containing 10, 20, and 40 mg of MWCNTs exhibited almost identical impedance magnitude over all frequencies when compared to pure hydrogel. Their slightly decreased conductivity is due to incorporated MWCNT, which has otherwise higher resistivity (80.7 Ohms, red spectrum) than pure electrolyte. When comparing pure electrolyte with the hydrogel containing 80 mg of MWCNTs, the most significant differences occurred in the mid and low frequencies, where interfacial and bulk ion diffusion is predominant.

The weakly resolved semicircles in impedance and admittance spectra (blue and green spectra, Figure 5A) indicate multiple circuit elements. Clearly, at higher MWCNT content, the two conductivity components can be separated. The one at higher frequencies is related to the more conducive hydrogel electrolyte and the second semicircle (at the middle range of frequencies) represents an electronic conductivity of the MWCNT fraction. This component is more clearly manifested in samples with 360 mg of MWCNT, with the resistance of MWCNT fraction that is very close to that of dry MWCNT (Figure 5, red). Yet, the total conductivity of the material is very similar to pure hydrogel over the broad range of MWCNT content. A significant change can be observed at the lowest frequency in both Bode diagrams. The magnitude of impedance decreases, and the phase angle changes from capacitive to diffusive (approaching −45°) for all hydrogels containing MWCNT. The Warburg is associated with a semi solid-state diffusion of ions in hydrogel. The Warburg coefficient representing transition time (*τ*, s) reflects the diffusion through the hydrogel is inversely related to the diffusion coefficient, according to Equation (1):

(1) $$\tau =\;L^{2}/D,$$

where *L* (m) is an effective diffusion length and *D* (m^2^ s^−1^) is a diffusion coefficient. The transition time (*τ*) is estimated based on fitting of an electrical equivalent circuit to the impedance spectra and obtaining Warburg resistor defined as:

(2) $$Z=\;\frac{Rctnh{\left(jT\omega \right)}^{P}}{{\left(jT\omega \right)}^{P}},$$

where *Z* is impedance, *ω* is frequency, *R* represents resistance, and *P* is a constant (0 < *P* < 1).

An effective diffusion length (*L*, m) is calculated using Equation (1) and the results are shown in Table 1. In general, the transition time *τ*, estimated from the fitting of the Warburg element, decreases with an increasing MWCNT content in the hydrogel. This increase for the electrode containing 360 mg of MWCNT is within two orders of magnitude of that of pure hydrogel. Consequently, an effective diffusion length for ion transport, *L*, decreases with increasing MWCNT amount. The trend in both, the time constant and the diffusion length, indicates that mass transport within the MWCNT-embedded hydrogels is improved as compared to the pure hydrogel. This can be rationalized as a positive effect of an increase of surface area between the electronic conductor (MWCNT) and the hydrogel electrolyte, resulting in the expansion of the double-layer interface within the material and the current collectors.

When the concentration gradient vanishes at the hydrogel/current collector interface, the Warburg impedance transforms into a capacitive behavior for pure hydrogel (black spectrum, Figure 5A) and for all samples with an MWCNT content lower than 80 mg (green spectrum, Figure 5A). This is demonstrated as an almost constant phase angle reaching a steady value of 80° for pure hydrogel. The low-frequency region is noticeably affected by the amount of MWCNT embedded in hydrogel. This is manifested by a change from capacitive behavior observed for pure hydrogel electrolyte to a more diffusive behavior for the MWCNT-hydrogel films. An additional diffusion process may occur at the MWCNT-hydrogel interface together with the surface diffusion of the adsorbed ions at the material/current collector. With increasing MWCNT content, this effect is stronger. Consequently, the low-frequency region of the impedance spectra for the film containing 360 mg of MWCNT shows an infinitive diffusion due to a large MWCNT-hydrogel interface across the material.

**Figure 6:**
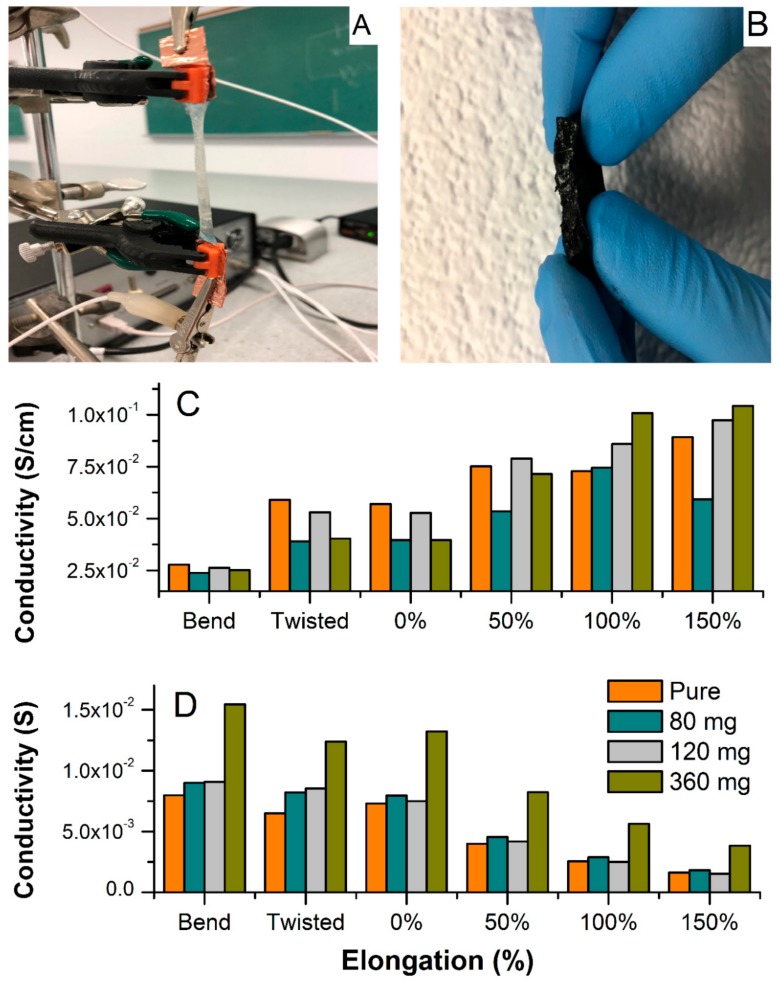
An electrochemical set-up for conductivity test upon elongation. (**A**) A cross-sectional photo of hydrogel-embedded MWCNTs (2.79 wt.% of MWCNT). (**B**) Total conductivity upon elongation, bending, and twisting normalized to the thickness, length, and width of the film upon elongation (**C**) and without normalization (**D**). The conductivity was estimated based on admittance analysis and example of spectra are shown in Figure 5A and Figure 7.

**Figure 7:**
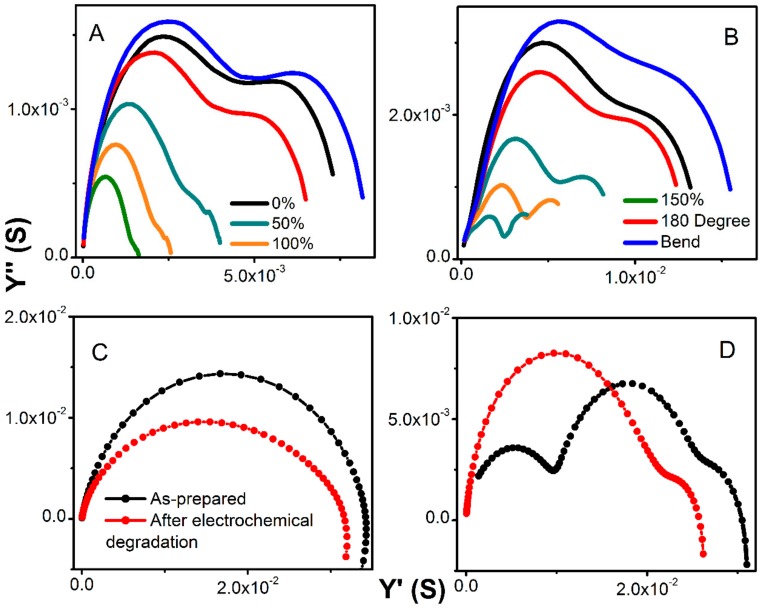
Admittance spectra upon elongation for pure hydrogel (**A**) and with 360 mg of MWCNT (**B**). Admittance spectra for as-prepared (black) and after a long-term charge-discharge (red) for pure hydrogel (**C**) and hydrogel with 360 mg of MWCNT (**D**).

Figure 6 shows the electrochemical set-up for testing conductivity upon elongation, bending, and twisting (A), and a cross-sectional photo of the MWCNT-hydrogel (2.79 wt.% of carbon), (B) together with a total conductivity of hydrogel electrodes and electrolytes normalized to the thickness, length, and width of the film upon deformation (C), and without normalization (D). Overall, the pure hydrogel electrolyte shows slightly higher specific conductivity as compared to the MWCNT-embedded films in steady state (Figure 6A). This decrease in total conductivity for the MWCNT-containing materials is because the resistance of pure MWCNTs is slightly higher than the hydrogel, as manifested in Figure 5A (red spectrum of bare MWCNT). However, the hydrogel with the highest MWCNT content has higher conductivity than the bare MWCNT. From the fitting of the admittance spectra, the conductivity of ionic fraction (hydrogel) and an electronic component (MWCNT) could be resolved and is included in Table 1. The ionic component (conductivity of pure hydrogel) is higher than the electronic one and remains constant regardless of the MWCNT content (5.2 × 10^−2^ S cm^−1^). The electronic counterpart evolves around values obtained for the bare MWCNT. A minor increase with increasing MWCNT content results in mixed conductivity that is close to that of pure electrolyte for the film containing 360 mg of MWCNT. This demonstrates that the hydrogel does not suppress MWCNT conductivity. The hydrogel matrix shows conductivity similar to liquid electrolyte and the MWCNT response is similar to what is typically observed in the liquid electrolyte with a similar KCl concentration [31]. All films showed no influence of twisting at about 180 degrees on the specific conductivity (Figure 6C,D). Furthermore, impedance was recorded during bending, and demonstrated a weak decrease when normalized to new geometries (Figure 6C), or no difference without normalization (Figure 6D). Interestingly, the elongation from original shape (0% elongation corresponds to as-prepared film) causes an increase in specific conductivity for all hydrogels since their thickness decreased significantly (although they were stretched to more than 150% of their original length; Figure 7A,B shows an evolution of admittance spectra upon elongation for pure hydrogels and with 360 mg of MWCNT). All electrodes demonstrated excellent reproducibility of electrical characteristics upon multiple stretching and deformation during repetitions of the same test (unless breaking was anticipated).

Yet, hydrogels with higher MWCNT content (240 and 360 mg, Figure 6C) showed improved total conductivity upon elongation when compared to pure hydrogel. This indicates that MWCNT not only generates electronic conductivity, but also enhances the total conductivity of the hydrogel, especially when subjected to mechanical deformations. This can be rationalized as improved tensile strength and rigidness of the MWCNT-embedded films. With that, some indication is given that weak chemical interactions between the MWCNT surface (enriched with oxygen-containing functionalities, i.e., carboxylic, phenolic, hydroxyl groups) and the acrylamide are possible, and result in improved mechanical and electrical characteristics of the combined materials. These molecular interactions can be also confirmed by the shift of onset peaks of hydrogel in the presence of MWCNT in the TGA spectrum (Figure 2). In addition, optical observations revealed a uniform distribution of MWCNT within the hydrogel, even at high MWCNT content (Figure 6B). The electrode shows good homogeneity and carbon dispersion across the film. This could be rationalized as both electrostatic and chemical effects i.e., related to the negative charge of MWCNT surface and alginate, and/or weak interaction between carboxyl groups present on the MWCNT surface with hydroxyl functionalities of alginate, resulting in chemical bond formation or possible electrostatic interaction. Thus, alginate not only participates in an ionic cross-linking of the base hydrogel matrix, but also modifies the MWCNT surface resulting in its better dispersion in aqueous solution, keeping in mind that these interactions are rather weak (hydrogen bridging or Van der Waal forces), unless esterification between these components is considered. At this point of the study, we can only speculate on the nature of these interactions since the FTIR did not show a significant difference between pure hydrogel and MWCNT-containing samples (Figure 4).

### 3.4. Electrochemical Stability

An accelerated charge-discharge led to degradation of the hydrogel at the point of contact of materials with the FTO current collector (manifested by slightly darker color at the hydrogel/FTO contact). Furthermore, TGA analysis of as-prepared and degraded hydrogels showed no differences in the thermograms, leading to conclusions that the increase in impedance (observed only at lowest frequencies) are related to the passivation of the FTO when exposed to corrosive components such as 1 M KCl for extended periods of time. Both the thermal and spectroscopic analysis have revealed that there is no noticeable chemical change within the material after long-term charging at 0.5 mA.

The total conductivity of hydrogel decreases only slightly and within the same order of magnitude as demonstrated on the admittance spectra in Figure 7C,D. Since the bare MWCNTs did not show any changes when subjected to charging at various current loads, we concluded that the decrease in conductivity is primarily due to the passivation of the contacts. The analysis of the Warburg element did not show any changes in time constant, or if any, the values laid within the calculation error. Yet, the decrease in admittance is slightly smaller for pure hydrogel as compared to the MWCNT-containing electrodes when tested in the long-term charge-discharge. This indicates slightly faster degradation due to the presence of MWCNT. Since the MWCNT enhances electrical contact between hydrogels, current collectors, and within materials, the degradation due to charging is accelerated.

**Figure 8:**
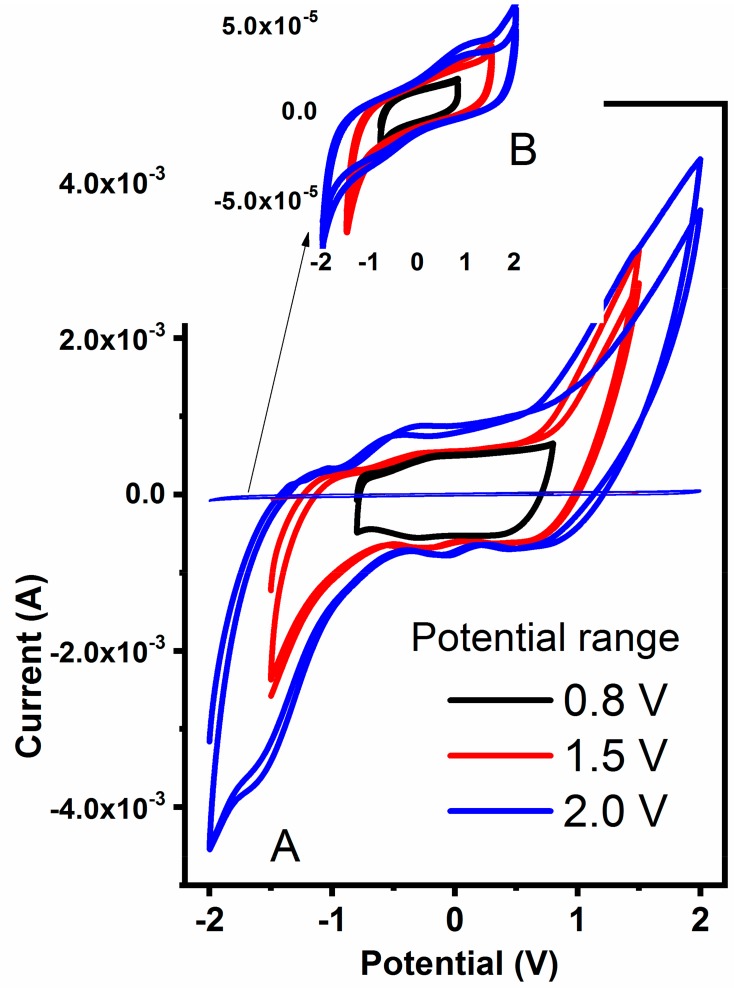
The cyclic voltammetry of the hydrogel containing 360 mg MWCNT (**A**) and pure hydrogel ((**B**), insert) scanned at various potential ranges at 0.2 V s^−1^.

To further estimate the practical potential windows for hydrogels, samples were polarized at various regimes and corresponding cyclic voltammograms are presented in Figure 8. In general, all hydrogels are stable within −0.95 and +0.95 V regardless of the MWCNT content, with a slightly improved potential stability for samples containing 360 mg of MWCNT. The materials are pronounced unstable above ±1.0 V due to water evolution as revealed by strong redox signals in CVs in Figure 8. An important observation was made on the current magnitude recorded for pure (Figure 8B, insert) and MWCNT-embedded hydrogel (Figure 8A). Due to the high content of MWCNT that are well-known to be double-layer capacitors, the current observed for combined hydrogel is two orders of magnitude higher when compared to pure hydrogel. Both the current and the shape of the CV spectrum indicate the strong capacitive nature of the latter, making them a potentially good candidate as an electrode material for flexible capacitor devices.

**Figure 9:**
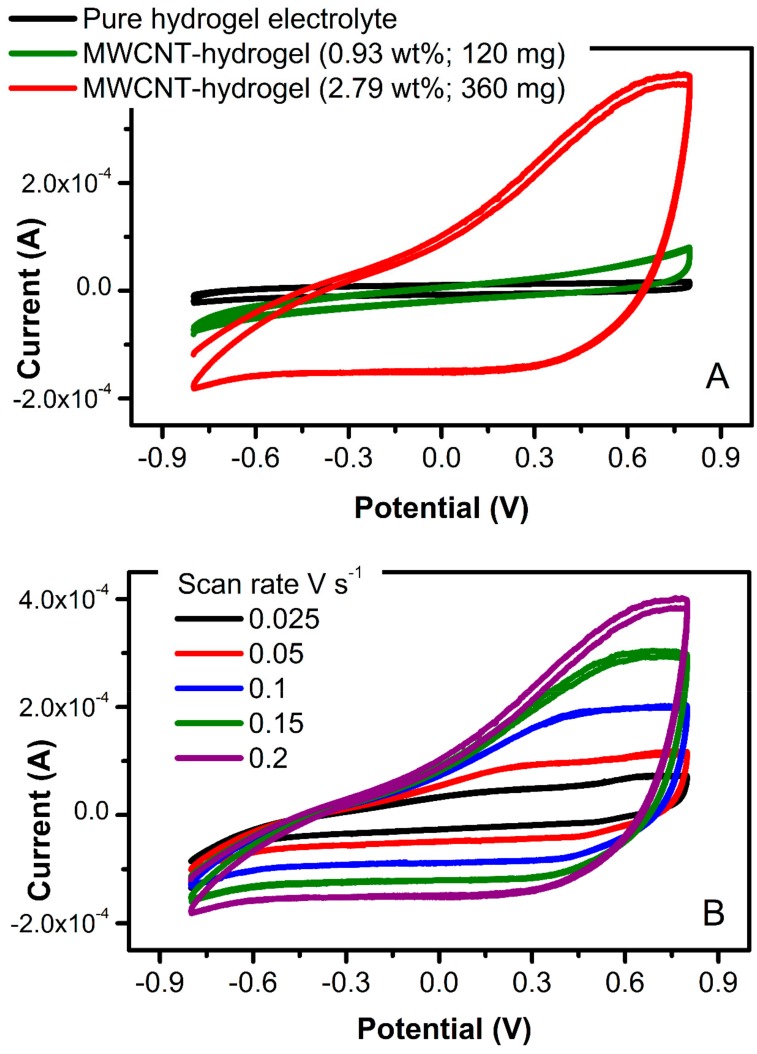
(**A**) CV scans recorded at 0.2 V s^−1^ of the pure hydrogel (black) and MWCNT-embedded hydrogel (green—0.93 wt.% and red—2.79 wt.% of carbon). (**B**) CVs of MWNT-embedded hydrogel (2.79 wt.%) at various scan rates.

Cyclic voltammetry (Figure 9A) was utilized to define the area-specific interfacial capacitance of the material (*C_s_*, F cm^−2^), calculated through Equation (3):

(3) $$C_{s}=\;\frac{1}{2}\frac{A_{CV}}{a∆V\nu }\prime$$

where *A_CV_* is the area bound by the CV (0.00284 AV), *a* is the electrode surface area (12.0 mm × 12.0 mm), Δ*V* is the potential window (1.6 V), and *ν* is the scan rate (0.2 V s^−1^). For the MWCNT-hydrogel at 3.69 wt.% of carbon, the area-specific interfacial capacitance was 27 mF cm^−2^. This interfacial double-layer capacitance of the carbon is within a range of the thin-film solid-state carbon electrodes (11 mF cm^−2^; [35]). The impact of carbon content on a magnitude of charging current has been noticed only at higher MWCNT concentration (higher than 240 mg of MWCNT, that is 1.88 wt.%; Figure 9A). This leads to the conclusion that the minimum carbon content of 2.0 wt.% is required in order to create flexible electrodes with desired electrical characteristics. Figure 9B demonstrates a typical increase of current with increasing potential scan rate (for the electrode with 2.79 wt.% of MWCNT), showing the ability of this electrode to pass more charge. The bulk capacitance of these electrodes is expected to be much higher and will be tested in a three-electrode electrochemical cell in a future study. An increase in charging current for the carbon-containing hydrogel in comparison to the pure hydrogel electrolyte shown in Figure 9A (black scan) indicates the viability of this conducting platform and its possible implementation in stretchable electronics.

## 4. Conclusions

We have investigated the effects of MWCNT addition within polyacrylamide-alginate hydrogels, on conductivity and mechanical characteristics in their steady state, and upon mechanical stretch. Based on the mechanical analysis, the tensile strength of pure hydrogel was similar to hydrogels reported in the literature, with increasing strength for MWCNT-embedded hydrogels synthesized in this work. The impedance spectroscopy revealed that the total resistance of electrodes decreased with increasing MWCNT content during their elongation, and that bending and twisting do not change their conductivity. The MWCNT-embedded hydrogels showed mixed ionic and electronic conductivities, both within the same range of 5–10 mS cm^−1^ in a steady state. In addition, the thermal stability of the electrode increased with increasing MWCNT content. This agreed with the long-term charge-discharge cycling that resulted in enhanced electrochemical stability of the MWCNT-hydrogel hybrid when compared to pure hydrogel electrolyte. These hydrogel films showed an interfacial double-layer capacitance at the high MWCNT content, which demonstrates their applicability in lightweight and yet flexible energy storage applications.

## Figures and Tables

**Table 1:** Time constant (*τ*) representing the transition time of an ion transport within a hydrogel obtained from the fitting of electrical equivalent circuits (Figure 5A, insert) and the conductivity of the MWCNT fraction incorporated into the hydrogel electrolyte. A conductivity of the hydrogel fraction in a steady state (without elongation) is 5 × 10^−2^ S cm^−1^, as demonstrated in Figure 6C.

Sample (mg of MWCNTs)	*τ*^1^ (s)	*L*^1^ (m)	Conductivity of MWCNT Fraction (S/cm)
0	5.72 × 10^−5^	1.61 × 10^−7^	-
20	1.53 × 10^−5^	8.36 × 10^−8^	-
40	2.17 × 10^−7^	9.95 × 10^−9^	0.014
80	1.82 × 10^−7^	9.12 × 10^−9^	0.010
120	1.14 × 10^−7^	7.21 × 10^−9^	0.014
240	4.18 × 10^−7^	1.38 × 10^−8^	0.015
360	3.36 × 10^−7^	1.22 × 10^−8^	0.023
Pure MWCNT	-	-	0.031

^1^*τ* = *L*^2^/*D* (*D* = diffusion coefficient estimated for 0.1 M KCl in acrylamide hydrogel as 4.57 × 10^−10^ m^2^ s^−1^ [34], *L* = an effective diffusion length for electrolyte species in acrylamide-based hydrogels.
